# When to settle for SETTLE! A lesson learned from our cases

**DOI:** 10.1186/s13044-023-00189-x

**Published:** 2024-03-04

**Authors:** Bangalore Rammohan Nagarjun, Shailee Mehta, Jahnavi Gandhi, Priti Trivedi, Priyank rathod

**Affiliations:** Present Address: Gujarat Cancer Research Institute (GCRI), Ahmedabad, Gujarat India

**Keywords:** SETTLE, Thyroid, Biphasic

## Abstract

Spindle epithelial tumor with thymic like elements (SETTLE) is a biphasic tumor composed of epithelial and spindle cell components. It is an uncommon indolent tumor arising in the thyroid gland and most commonly affects the children and young adults. This entity is mostly overlooked because of its rarity and diagnostic difficulty on morphology. We discuss two cases of SETTLE with varied presentation, diagnostic challenges and lessons learnt from them.

SETTLE should be considered as a differential especially when dealing with a thyroid lesion in young and adolescent. The article discusses the histologic details and common mimickers to be borne in mind aiding in arrival at the final diagnosis on biopsy specimens.

## Introduction

Spindle epithelial tumor with thymic -like elements (SETTLE), is a rare tumor arising in the thyroid gland. It is usually a biphasic tumor, described as “a compact arrangement of spindle and epithelial cells” [1] Embryonically, this tumor is believed to arise from remnants of the brachial pouch or thymus. It mostly affects children and young adults. SETTLE is an indolent, slow-growing tumor with normal thyroid functions [2, 3]. Ultrasound shows a mixed solid-cystic lesion that is cold on thyroid scinti-scanning [4–6]. When surgically removed completely, it confers a good prognosis. However, reports of distant metastasis and local recurrence have been documented, but the true malignant potential of the tumor is not well established due to a limited number of long term follow-up of cases diagnosed with this rare entity [2].

SETTLE pathologically mimics epithelial and mesenchymal tumors; therefore, the diagnosis of SETTLE can be overlooked. Here we present two cases and discuss their morphologic mimickers. It is important to distinguish these from other morphologic mimics due to therapeutic and prognostic implications.

### Case 1

**Fig. 1:**
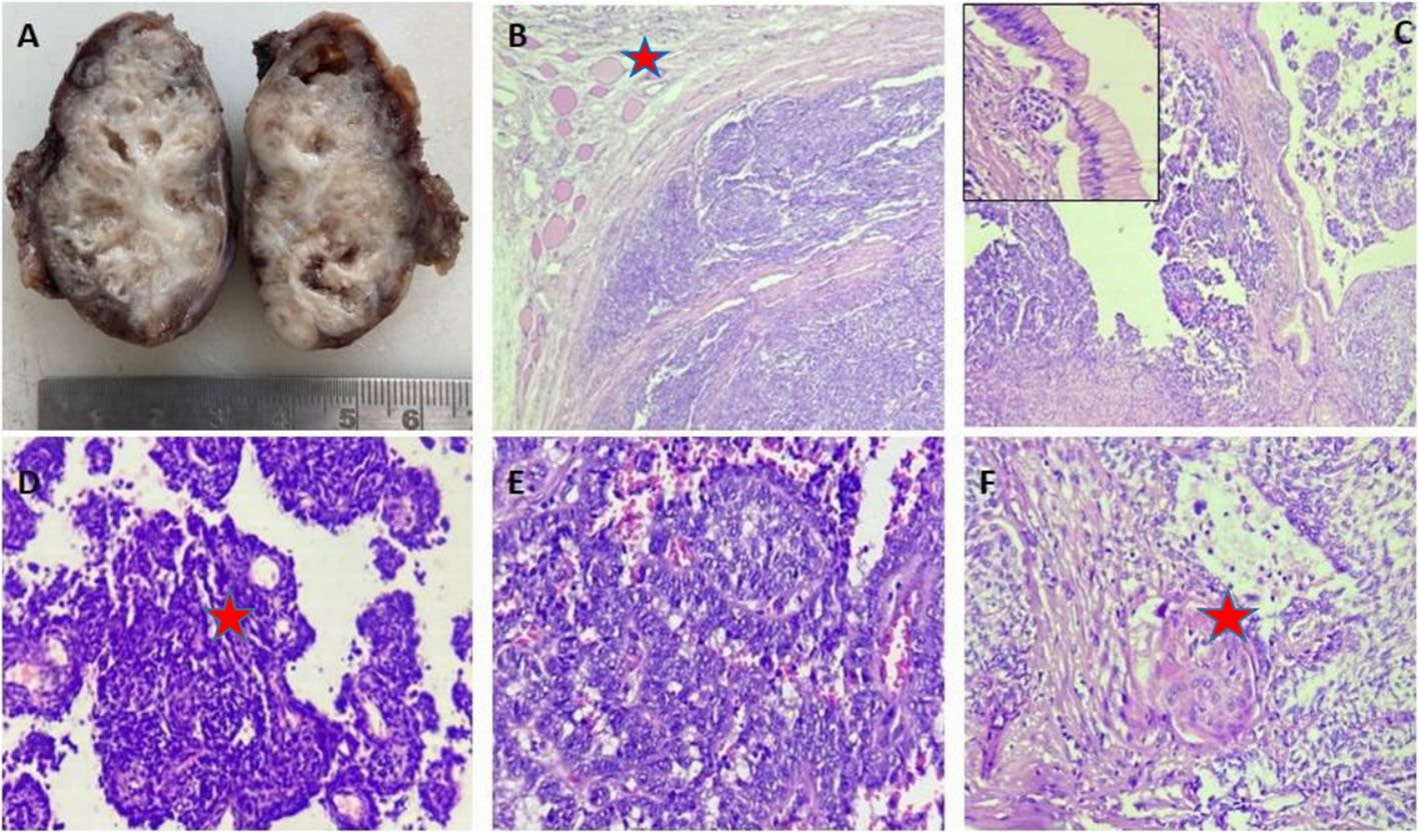
Gross of the cervical node shows predominantly solid and focal cystic area. The tumor appears tan white and firm in consistency. **A** Shows circumscribed tumor with thyroid parenchyma at periphery × 40. **B** inset show epithelial component lined by columnar epithelium. **C** Tubulopapillary architecture of the tumor is evident × 100. **D**,**E** Squamoid nest is noted × 400. **F**

**Fig. 2:**
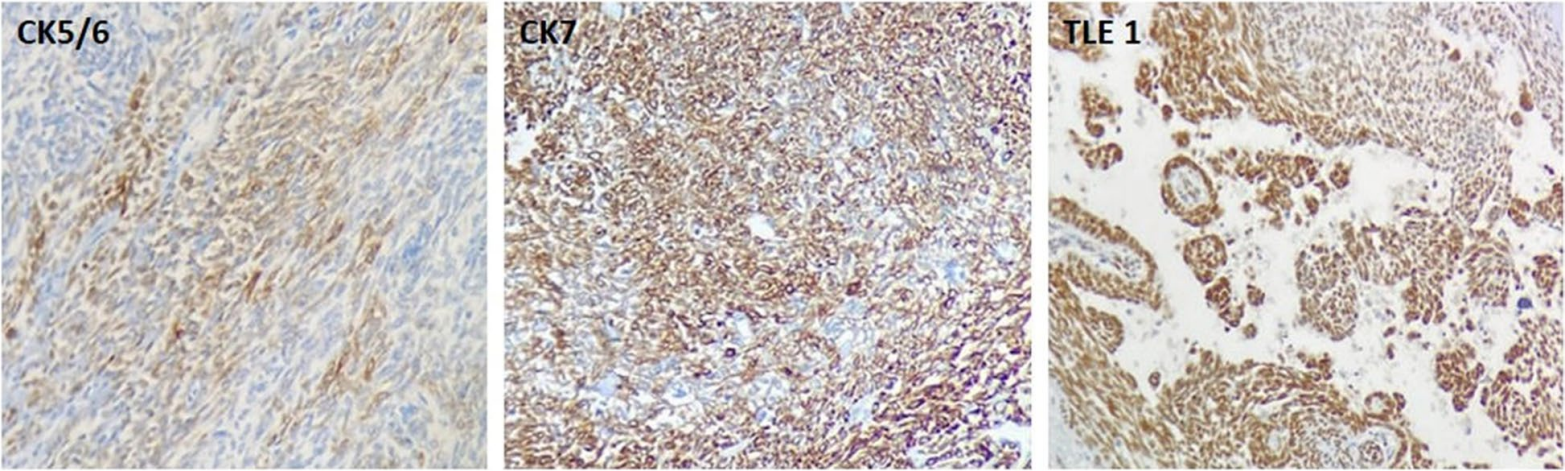
CK5/6 stains with moderate intensity noted focally in the spindle cell component. **A** CK 7 and TLE1 show moderate to strong staining intensity with diffuse pattern in epithelial and spindle cell component. **B**, **C**

A 14-year-old male presented with an enlarged left level V node. Contrast-enhanced computed tomography (CECT) imaging showed a well-defined necrotic node measuring 1.7 × 2.3x4.9 cm. The patient had a history of left hemithyroidectomy 18 months back for a neck swelling of one year duration. Trucut biopsy of the node revealed a biphasic tumor with bland appearing spindle cells arranged in fasicular pattern and epithelial component in tubulopapillary pattern..On reviewing the hemithyroidectomy slides they revealed a well circumscribed lobulated tumor with a biphasic pattern, composed predominantly of spindle and epithelial components. The epithelial components had focal glandular and squamoid components. The spindle cell component was characterized by shohttps://www.msn.com/en-ph/feedrt fascicles.The epithelial component had tumor cells arranged in papillary pattern with hyalinized to fibrosed areas. Normal thyroid parenchyma was noted at the periphery. (Image 1) There was no evidence of necrosis or mitosis. Immunohistochemistry (IHC) showed pan-cytokeratin (AE1/AE3), high-molecular weight cytokeratin (CK5/6), CK7, and vimentin positivity in the epithelial component and spindle cell component. The spindle cell component was also positive for smooth muscle actin (SMA) and TLE1(Image 2)and immunonegative for CD34, S100, TTF1, CD5, CD117, calcitonin, CD99, and BCL2. Considering the overall findings, the diagnosis of metastatic SETTLE was favoured and left modified neck dissection was performed (Figs. 1 and 2).

### Case 2

A male patient aged 58 years with a history of recurrent neck swelling had undergone total laryngectomy with radical neck dissection. Slides were submitted for second opinion. CT findings revealed soft tissue density lesion eroding the thyroid cartilage and abutting the cricoid. The patient had a significant past history of being operated twice for thyroid swelling. 20 years ago, he underwent right hemithyroidectomy for papillary carcinoma thyroid and 10 years later, presented with nodal recurrence. Then he underwent completion thyroidectomy and radioiodine therapy. On reviewing the slides, a characteristic finding of biphasic tumor with epithelial and spindle cell components, abutting thyroid and cricoid cartilage was observed.. IHC results showed both the components immunoreactive for AE1/AE3, CK5/6, and vimentin. Tumor cells were immunonegative for CD99, BCL2, thyroglobulin, TTF-1, PAX 8, CD 5 and was finally diagnosed as SETTLE. Presently the patient is on adjuvant radiotherapy.

## Discussion

SETTLE, a less known entity, was christened by Chan and Rosai in 1991 [7]. Early cases were reported under different designations, such as an “unusual thyroid tumor in a child,” “thyroid spindle cell tumor with mucous cysts,” “thyroid thymoma in childhood,” or malignant teratoma of thyroid [2]. With the concept of SETTLE, nearly 50 cases have been documented till 2021 based on pubmed search.

This neoplasm is commonly observed in children, adolescents, and young individuals. Few reports of old age have been documented. The male-to-female ratio is 1.5:1 and the average size ranges between 0.5 and 12 cm [8–12].

SETTLE commonly presents as a painless mass in the neck but symptoms such as superior vena caval syndrome, tracheal compression or paraneoplastic hypercalcemia are also noted [2, 13]. Most investigations such as thyroid function test, calcitonin are normal. Ultrasonography is not characteristic, but a hypoechoic or heterogeneous solid-cystic pattern and cold thyroid scans have been reported [4–6].

Fine-needle aspiration is rarely diagnostic [14]. The cytological features of SETTLE have been reported as a highly cellular aspirate composed mainly of cohesive or isolated sheets of spindle cells [8, 15, 16]. Nuclear pleomorphism is unusual and mitotic figures are uncommon. Spindle cells are often embedded in metachromatic extracellular matrix in air-dried diff quick stained smears which raises, possibility of medullary carcinoma, but with normal calcitonin and CEA levels, suspicion of SETTLE should be raised.

Preoperative diagnosis of this entity is difficult because of the absence of specific clinical symptoms, radiologic findings, or serum markers. It is often misdiagnosed and mistreated as medullary carcinoma thyroid, teratoma, or papillary carcinoma thyroid [1, 2, 14, 17–19].

**Fig. 3:**
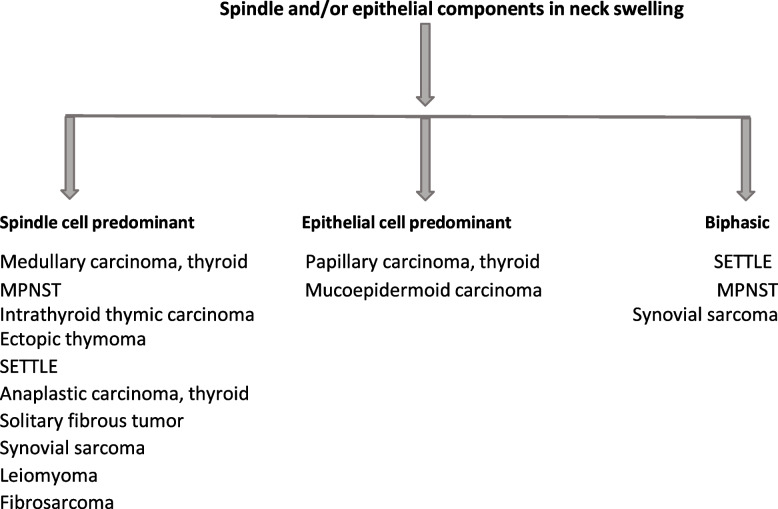
The chart enumerates the possible differentials to be considered based on the histologic component noted

This review discusses histological pearls and pitfalls to be considered for the possibility of SETTLE on biopsy based on our own experience and cases Fig. 3.

SETTLE morphologically shows a lobular pattern divided by incomplete fibrotic septae with encapsulated to infiltrative margins. The lobules show a biphasic pattern with spindle and epithelial components which blend imperceptibly. The epithelial component show cords, tubules, papillae, or glandular patterns. Multiple differentials are considered based on the predominant component sampled on biopsy. 1) spindle, 2) epithelial or 3) mixed.

### Spindle cells are predominant

The spindle cell variant of medullary thyroid carcinoma resembles mesenchymal neoplasm with monomorphic spindle cells arranged in fascicles seperated by delicate fibrovascular septa. The thick fibrotic bands seen in SETTLE may mimic amyloid-like material. Congo red, serum, and immune markers such as calcitonin and CEA help to confirm the diagnosis [20].

When the spindle cell component is noted, arranged in a fasicular pattern with round to oval nuclei, fine chromatin, pointed ends, minimal pleomorphism, inconspicuous nucleoli, scant to moderate amount of cytoplasm, synovial sarcoma is the commonest differential to be considered. Clinical history of rapid increase in size and aggressive behavior will be helpful in distinguishing it from SETTLE. TLE 1 positivity is a potential pitfall as SETTLE expresses moderate to strong positivity, as in our case. Fluorescent in situ hybridization (FISH) for t (x;18) or PCR will be of prime value in challenging cases [21, 22].

Another common differentials are tumors of neural origin ranging from benign neural to malignant nerve sheath tumor (MPNST). Certain morphological findings, such as irregular wavy buckled nuclei, dense marbling cellular to hypocellular areas, myxoid and fibrillary background point towards neural tumor. S100 and SOX10 further aid in deciphering neural differentiation.

Ectopic thymoma, type A is considered as a differential. The epithelial cells show spindle cell morphology with fasicular, storiform pattern, bland nuclei with dense chromatin and indistinct nuclei. Sometimes areas of glandular, papillary, pseudo-papillary, or cyst formation can be seen. On IHC, epithelial cells stain for CK, EMA, and p40 and also stain for B cell marker CD20. Scattered lymphocytes, if present show mature phenotype with CD3 positive and Tdt negative.

For practical purposes, any thyroid tumor resembling a sarcoma, especially in the elderly, anaplastic carcinoma should be considered. Histomorphologically, it shows nuclear pleomorphism, areas of necrosis and/or mitosis and also, the clinical history of rapid increase in size of neck swelling is very helpful. PAX8 staining (seen in 36–76% cases) [23], TTF1 and focal keratin positivity also helps in confirming an anaplastic carcinoma of thyroid [24, 25].

Solitary fibrous tumor rarely occurs in the thyroid gland and shows round to fusiform cells with indistinct cell borders arranged around ramifying blood vessels with areas of hyalinisation. CD34 and STAT6 positivity confirm the diagnosis [26].

Leiomyoma is an extremely rare entity in the thyroid but worth mentioning. This tumor shows fascicles of spindle cells with cigar shaped nuclei and an abundant amount of eosinophilic cytoplasm. The tumor sometimes shows positivity for SMA along with CK, which is seen as a potential pitfall in the diagnosis of SETTLE as found in our case. Hence, inclusion of desmin is necessary before considering leiomyoma [27].

A rare variant of papillary thyroid carcinoma (PTC) with a prominent stromal component and small foci of PTC is called PTC with exuberant nodular fasciitis-like stroma. The stromal component resembles the appearance of nodular fasciitis or fibromatosis of the soft tissues. It is Diagnosed only after thorough sampling to detect the epithelial component [28].

The remote possibility of fibrosarcoma of the thyroid, which is generally seen in older age, is to be considered when IHC findings are inconclusive and morphology reveals intersecting fascicles of spindle cells [29].

The possibility of primary or metastatic melanoma should be considered in differential as it is notorious for its atypical presentation and markers such as Melan A, HMB45, MITF, S100 are helpful in clinching the diagnosis [30].

### The epithelial component is predominant

The epithelial component was a minor component in both of our cases. However, on biopsy when the sampled component is mostly epithelial in nature, differentials considered is metastatic adenocarcinoma when glandular component is seen. Mucoepidermoid carcinoma is a possibility when squamoid areas or cells with cytoplasmic mucin are observed. The foamy and clear cells are positive for periodic acid schiff and mucicaramine stain [31]. Epithelial components sometimes raise a possibility of teratoma and however, diligent search for all three germ layers is diagnostic.

Tubulopapillary architecture on biopsy is a potential pitfall and is generally misdiagnosed as papillary thyroid carcinoma, as was noted in one of our cases. However, a strict vigil for characteristics nuclear features such nuclear overlapping, grooving, nuclear clearing with ground glass or fine chromatin will avoid misdiagnosis. Tumor cells with vesicular nucleus, indistinct cell borders or squamoid nature mimic intrathyroid thymic carcinoma (formerly called CASTLE) but CD5 and CD117 markers are almost always positive.

### Spindle and epithelial components in varied proportions

Biphasic synovial sarcoma is composed of epithelial components which are cuboidal to columnar cells. Arranged in a glandular or solid pattern and intimately associated with malignant spindle cell component. Markers such as TLE 1, CD 99, BCL 2, CD 34 are expressed in both SETTLE and synovial sarcoma. Both morphology and IHC they mimic very closely. Clinical presentation of progressive increase in size and FISH analysis helps in definitive diagnosis. However, primary synovial sarcoma of thyroid is extremely rare with very few case reports [21, 32].

Malignant peripheral nerve sheath tumor with glands occurs in the younger population and is associated with major nerves. Histomorphologically shows a glandular component composed of columnar to cuboidal cells with clear cytoplasm and occasionally goblet cells along with spindle cells component. Heterologous elements such as cartilage and bone are reported [32].

Thyroidectomy is the mainstay of treatment, lobectomy or total thyroidectomy is based on the extent of disease. Two cases have been reported where separate foci of SETTLE were noted in the retained lobe of thyroid gland years after first diagnosis [8]. Lymph node dissection is performed only when nodes are involved or suspicious of metastasis. Reports of good outcome are noted at surgically amenable metastatic sites as well. Response to adjuvant radiotherapy and chemotherapy in inoperable and metastatic cases are documented. Various chemotherapy regimens have been tried on patients with SETTLE. Chemotherapy has been administered in adjuvant and metastatic setting. Response to therapy in the limited number of cases is varied from stable disease to cases having partial to complete response. Response to radiotherapy has also been reported [2].

Since the incidence of tumor is rare, risk factors associated with its recurrence are not known. Nevertheless, certain risk factors are proposed such as extrathyroidal extension, lymphovascular invasion, lymph node metastasis or infiltrative borders however, more data is required for definite opinion [3].

SETTLE is a slow growing neoplasm with metastasis in approximately 26% of cases. Since the tumor is generally slowly growing and indolent in nature, patients seek delayed medical attention which could result in metastasis [2]. The principal site of metastasis is lung, which represents more than 60% of the metastasis [33–35]. Other reported sites include mediastinum, regional lymph nodes, liver, kidney, pancreas, bone, pleura and peritoneum. Local recurrence in the tumor bed is also documented [2]. The period between diagnosis and detection of metastasis varies from a few months to two–three decades [36, 37]. Owing to the delayed metastasis, it is advised to perform radiological investigations to look for local/ distant disease, despite the absence of specific symptoms [13].

Diagnosis of this entity is essential as it requires the patient to be on regular long term follow-up for detection of metastasis [13]. The metastatic potential is difficult to assess. Cheng et al. suggested metastatic risk increases and is as high as 71% over a follow-up period of five years or more. Only with more data, the absolute risk of metastasis can be established as discussed in our two cases. Case 1 had metastasis as early as one year. Case 2 presented twice with metastasis, first was seen 10 years from initial diagnosis and again six years after the first metastasis.

SETTLE, which is described as a low grade malignant tumor with a favourable prognosis after complete surgical resection, can have an aggressive course in some cases [5]. SETTLE patients have long survival despite the development of metastasis [38].

## Conclusion

SETTLE,is a rare tumor occurring in the thyroid gland.Lack of awareness of this entity makes pre-operative diagnosis challenging. Awareness of the histologic pattern especially in small biopsies, along with support of IHC is the key to diagnosis. This entity should be considered in the list of differential in neck swelling particularly in children and young adults. Apt pre-operative diagnosis and appropriate surgical resection with clear margins is the mainstay which helps to reduce recurrence/metastasis. and prolong patient survival.

It is extremely important to differentiate it from biphasic synovial sarcoma, which is a close mimicker, as both share similar morphology and IHC features but have different management and outcome. A multidisciplinary approach is advisable in such cases as the treatment modality and prognosis differs.
